# Primary varicella-zoster virus infection of the immunocompromised associated with acute pancreatitis and hemophagocytic lymphohistiocytosis: A case report

**DOI:** 10.1097/MD.0000000000025351

**Published:** 2021-04-23

**Authors:** Antoine Bérar, Samuel Ardois, Patricia Walter-Moraux, Marc-Antoine Jegonday, Basile Henriot

**Affiliations:** a CHU Rennes, Department of Internal Medicine and Clinical Immunology, Rennes; b CH René Pleven, Department of Gastroenterology and Hepatology, Dinan; c CHU Rennes, Department of Radiology, Rennes; d CH René Pleven, Department of Internal Medicine, Dinan; e CH Broussais, Department of Internal Medicine, Saint-Malo, France.

**Keywords:** hemophagocytic lymphohistiocytosis, pancreatitis, varicella zoster virus infection

## Abstract

**Rationale:**

Primary varicella-zoster virus (VZV) infection may be associated with hemophagocytic lymphohistiocytosis (HLH), as well as with acute pancreatitis. However, there is few data concerning the evolution and the optimal treatment of these rare associations.

**Patient concerns:**

A 57-year-old immunocompromised woman, who was treated for chronic lymphocytic leukemia 3 years prior to admission, was hospitalized with abdominal pain revealing severe acute pancreatitis. The day after admission, a pruritic rash appeared on her face, trunk, and limbs, sparing the palmoplantar regions. At the same time, fever, thrombocytopenia (27 × 10^9^/L), major hyperferritinemia (11,063 μg/mL), hypertriglyceridemia (2.56 mmol/L) and elevated lactate dehydrogenase levels (1441 IU/L) suggested HLH.

**Diagnosis:**

The diagnosis of chickenpox (varicella) was established. Primary VZV infection was then confirmed: cutaneous and plasma VZV polymerase chain reactions were positives, VZV serology was negative for IgG.

**Interventions:**

Treatment with aciclovir was started intravenously after the onset of the rash, for a total of 10 days. A 48-h surveillance in intensive care was carried out.

**Outcomes:**

Acute pancreatitis and biological abnormalities evolved favorably under aciclovir. Platelet count was normalized 6 days after admission to hospital.

**Lessons:**

A favorable outcome of primary VZV infection associated with severe acute pancreatitis and probable HLH in an immunocompromised patient is possible with aciclovir alone.

## 1 Introduction

The association between primary varicella zoster virus (VZV) infection and hemophagocytic lymphohistiocytosis (HLH), formerly known as macrophage activation syndrome, has already been described in adults.^[1–3]^ Similarly, the association between primary VZV infection and acute pancreatitis is rare but known, including in immunocompromised adults.^[3–6]^ However, the evolution and optimal treatment of these presentations are poorly defined. We report a case of varicella, severe acute pancreatitis and probable HLH in an immunocompromised patient.

## 2 Case report

A 57-year-old woman was admitted to hospital with abdominal pain, nausea and vomiting that had progressed over the past 6 days. She had a prior history of chronic lymphocytic leukemia treated with rituximab, fludarabine, and cyclophosphamide 3 years prior to admission, and considered in complete remission. Subsequent hypogammaglobulinemia at 3 g/L had been compensated for by intravenous immunoglobulin substitutes up to 1 year after the end of treatment. Her only long-term treatment was eye drops (latanoprost).

**Figure 1:**
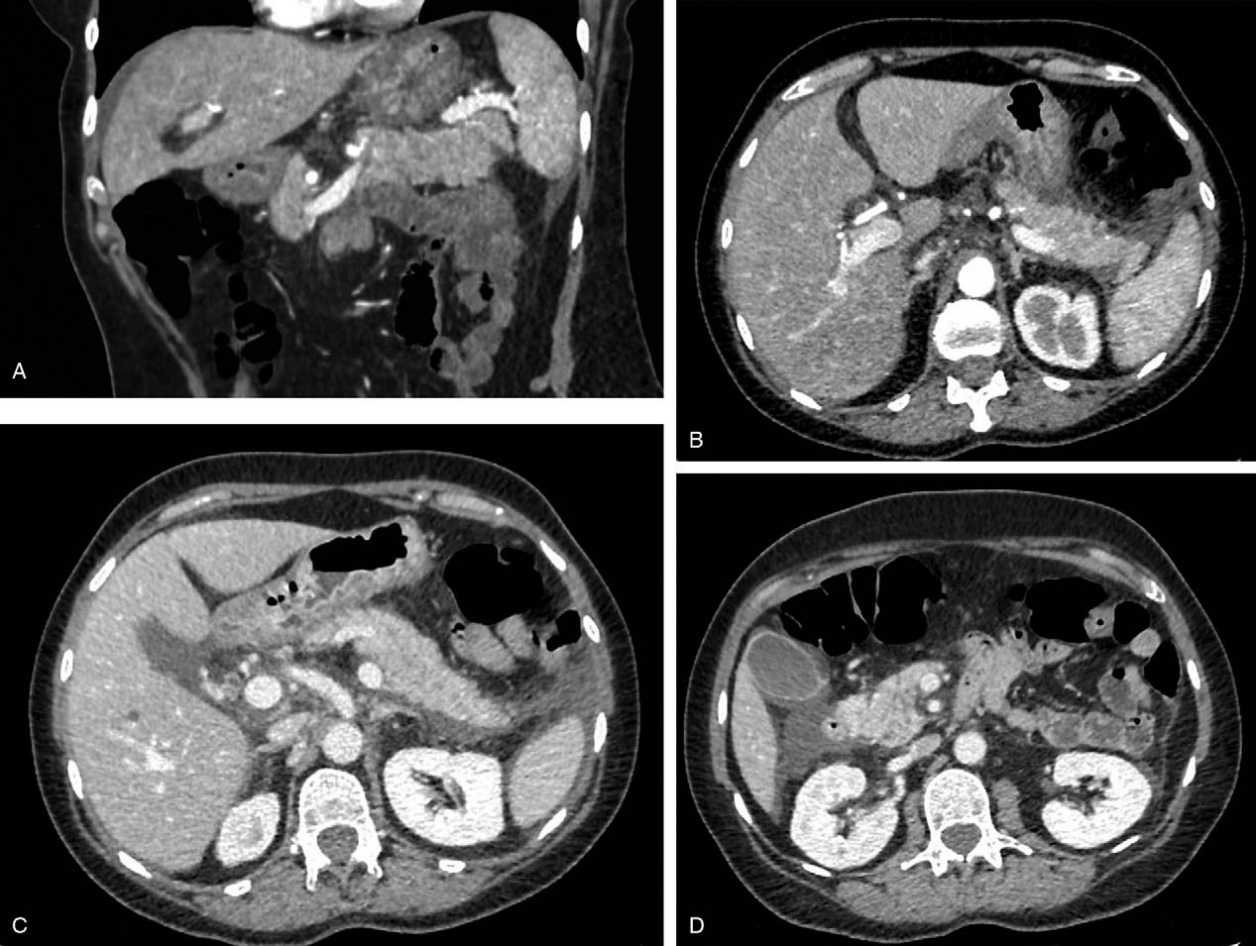
Abdominal CT: area of pancreatic parenchymal necrosis (hypodense notch of the tail of the pancreas <30%) in coronal section (A) and in axial section (B), peri-splenic necrosis casting also in front of the left anterior para-renal fascia (C), necrosis casting also in front of the right anterior para-renal fascia (D).

Physical examination showed a painful abdominal palpation without signs of peritoneal irritation. Lipasemia was 252 IU/L, C-reactive protein 40 mg/L, aspartate aminotransferase (AST) 720 IU/L, alanine aminotransferase (ALT) 926 IU/L, total bilirubin 22 μmol/L. Abdominal-pelvic computed tomography (CT) revealed acute pancreatitis associated with two necrotic lesions (Balthazar E score, CT severity index 5) (Fig. 1). There was no biliary lithiasis on ultrasonography. There was no hypercalcemia. The patient reported consuming two units of alcohol per day. A pancreatitis of alcoholic origin was initially considered.

**Figure 2:**
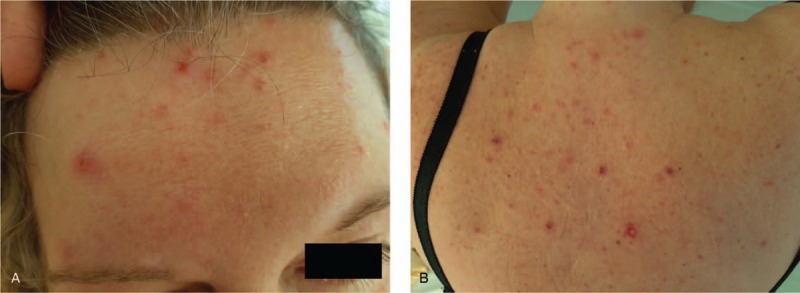
Vesicular skin rash of the face (A) and back (B).

The day after admission, a pruritic rash appeared on her face, trunk, and limbs sparing the palmo-plantar regions, for which the diagnosis of varicella was made (Fig. 2). To the best of her knowledge, the patient had never developed this condition. Concomitantly, cellularity appeared on complete blood count. Treatment with aciclovir was started the same day intravenously (15 mg/kg every 8 h).

Platelet count decreased in one day from 82 × 10^9^/L to 27 × 10^9^/L. There was no other cytopenia or evidence of disseminated intravascular coagulation. New biological assessment showed hyperferritinemia (11,063 μg/mL), hypertriglyceridemia (2.56 mmol/L) and elevated lactate dehydrogenase (1441 IU/L). Fever was also noted. There was no hepatosplenomegaly.

The HScore, used to estimate the risk of HLH, was 164, meaning a probability of having a HLH of 44.5%.^[7]^ In view of the potential severity of this syndrome, specific anti-VZV immunoglobulins and specific treatments for HLH were discussed on the basis of the literature.^[8,9]^ Finally, we did not perform a therapeutic intensification because of the patient's preserved general state and a tendency to improve on a close biological evaluation. The hepatic function remained conserved. A 48-h intensive care monitoring was performed. HLH markers progressively improved: triglyceridemia, AST and ALT decreased, and platelet count increased (45 × 10^9^/L). Consequently, no myelogram was performed.

Skin and plasma varicella zoster virus (VZV) polymerase chain reactions (PCR) were positive. Epstein–Barr virus (EBV), cytomegalovirus (CMV) and herpes simplex virus plasma PCR were negative. VZV serology was negative for IgG. EBV, CMV, and toxoplasmosis serologies were in favor of acquired immunity. Hepatitis A virus, hepatitis B virus, hepatitis C virus, hepatitis E virus, and human immunodeficiency virus serologies were negative.

The diagnosis of primary VZV infection was thus confirmed and treatment with aciclovir was persued for a total of 10 days. Aciclovir was well tolerated. The evolution of the rash and abdominal pain was favorable within a few days. Six days after admission to hospital, platelet count was normalized. On the other hand, exocrine pancreatic insufficiency persisted following acute pancreatitis. An abdomino-pelvic CT performed one month after the onset of symptoms showed the disappearance of pancreatic necrosis and abdominal necrosis flows.

Intravenous immunoglobulin were reintroduced considering the severe infectious episode and persistence of profound hypogammaglobulinemia (3.4 g/L). Gammaglobulin rate normalized and no further infectious episodes occurred since then.

## 3 Discussion

We present a primary VZV infection in an immunocompromised patient characterized by acute pancreatitis followed by the appearance of a typical chickenpox rash and a possible HLH. Delayed onset of rash compared to the first symptoms of acute pancreatitis has been reported elsewhere in cases of VZV infection in immunocompromised patients.^[3,5,6]^

The hypothesis of HLH was based on underlying immunosuppression, fever, thrombocytopenia, elevated transaminases and hyperferritinemia. Most of these signs could be related to a cause other than HLH: fever could be explained by varicella or acute pancreatitis; thrombocytopenia could also be related to varicella; and elevated transaminases could be related to varicella through viral biological hepatitis.^[10]^ In contrast, the elevation of ferritinemia to more than 5000 μg/L points towards HLH, since its main causes other than HLH are only adult Still's disease and certain malignant hematological diseases.^[11]^

Prevention of primary VZV infection can be considered by various means. Given the theoretical contraindication to live attenuated VZV vaccination in immunocompromised patients, some authors have proposed generalized prophylaxis with valaciclovir in this category of patients not immunized against VZV.^[5]^ Vaccination could nevertheless be proposed, taking care to administer it before starting chemotherapy, if there is no history of varicella and if serology is negative. In addition to the live attenuated vaccine (Zostavax), a more effective recombinant subunit adjuvant vaccine (Shingrix) is already available in some countries.^[12]^ The vaccination of the immunocompetent household members of patients is an alternative or complementary strategy that could also be considered, although in our case, no case of varicella in the entourage has been known.

## Author contributions

**Conceptualization:** Basile Henriot.

**Resources:** Antoine Berar, Marc-Antoine Jegonday, Basile Henriot.

**Supervision:** Basile Henriot.

**Validation:** Antoine Berar, Samuel Ardois, Patricia Walter-Moraux, Marc-Antoine Jegonday, Basile Henriot.

**Visualization:** Antoine Berar.

**Writing – original draft:** Antoine Berar.

**Writing – review & editing:** Antoine Berar, Samuel Ardois, Basile Henriot.
